# Changes in the anti-inflammatory activity of soy isoflavonoid genistein versus genistein incorporated in two types of cyclodextrin derivatives

**DOI:** 10.1186/1752-153X-6-58

**Published:** 2012-06-20

**Authors:** CorinaTiulea Danciu, Codruta Soica, Erzsebet Csanyi, Rita Ambrus, Stefana Feflea, Camelia Peev, Cristina Dehelean

**Affiliations:** 1 Department of Pharmacognosy, “Victor Babes” University of Medicine and Pharmacy, 2 EftimieMurgu, Timisoara 300041, Romania; 2 Department of Pharmaceutical Chemistry, “Victor Babes” University of Medicine and Pharmacy, 2 EftimieMurgu, Timisoara 300041, Romania; 3 Department of Pharmaceutical Technology, University of Szeged, 6 Eotvos u., Szeged, H-6720, Hungary; 4 Department of Toxicology, “Victor Babes” University of Medicine and Pharmacy, 2 EftimieMurgu, Timisoara 300041, Romania

**Keywords:** Genistein, Cyclodextrins, Inflammation, Haematoxylin-eosin, CD45, C57BL/6 J mouse

## Abstract

**Background:**

The isoflavonoid genistein represents the major active compound from soybean, the vegetal product from *Glycine max (Fabaceae)*. The aim of this study is to prove that genistein was incorporated in two semisynthetic cyclodextrins, beta-cyclodextrin derivatives: hydroxypropyl-beta-cyclodextrin and randomly-methylated-beta-cyclodextrin as well as to compare the anti-inflammatory activity of genistein with that of genistein incorporated in these two types of semisynthetic cyclodextrins.

**Results:**

The animal studies were conducted on 8-week old C57BL/6 J female mice. Inflammation was induced in both ears of each mouse by topical application of 10 micrograms 12-O-tetradecanoylphorbol-3-acetate dissolved in 0.1 ml solvent (acetone : dimethylsulfoxide in a molar ratio 9:1). Thirty minutes later treatment was applied. The inflammatory reaction was correlated with increased values in ear thickness. Treatment with genistein and genistein incorporated in the two cyclodextrins led to decreased values for ear thickness. Better anti-inflammatory action was found for the complexes of genistein. Both haematoxylin-eosin analysis and CD45 marker expression are in agreement with these findings.

**Conclusions:**

Results allow concluding that genistein is an active anti-inflammatory phytocompound and its complexation with hydrophilic beta-cyclodextrin derivatives leads to a stronger anti-inflammatory activity.

## Background

Flavonoids (*flavus* = yellow) are a class of secondary plant metabolites which function mainly as vegetal pigments, as antibiotic defence substances and as signal molecules for beneficial micro organisms in the rhizosphere [1]. Researchers continue to find new functions for this class of compounds, such as components of a healthy human diet [1,2].

**Figure 1:**
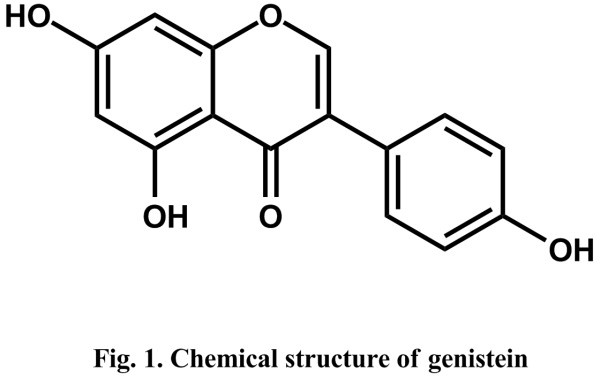
Chemical structure of genistein.

The isoflavonoid genistein (Gen) (4’, 5, 7-trihydroxyisoflavone) (Figure 1), the aglycone of the heterosidegenistin, represents a major active compound from soybean [2], the vegetal product from *Glycine max* (*Fabaceae)*. Previous studies have demonstrated that genistein possesses many biological functions, such as preventive of coronary heart disease and osteoporosis, antioxidant, antineoplasic and anti-inflammatory compound [3-6].

Inflammation is a localized reaction of tissues in response to an aggressive action, characterized by redness, warmth, swelling, pain, and sometimes loss of mobility or function. Inflammation is considered a beneficial and necessary attempt of the organism to eliminate the aggressive agent and to start the healing process, which can consist in suppression of tumour initiation or progression. As a consequence, when the control mechanism of inflammation does not function properly and the inflammation persists, diseases including cancer may develop [7,8]. Previous studies have demonstrated that genistein and other flavonoids exhibit an anti-inflammatory effect both for human and mouse skin, as well as inhibitory action against the activation of nuclear factor-kB and secretory phospholipase A2 [9-11].

Genistein shows a high solubility in organic solvents such as dimethylsulfoxide (DMSO), dimethylformamide, acetone and ethanol. Due to its chemical structure, it has, however, a very poor solubility in water, which drastically reduces its bioavailability [12]. Inclusion complexes are nowadays largely used in the pharmaceutical field in order to optimize the solubility, chemical stability and bioavailability of guest molecules. Recently, much interest has focused on cyclodextrins (CD), because of their remarkable ability to form host–guest inclusion complexes with a wide variety of molecules, changing the physical-chemical properties of the guests [13,14]. β-cyclodextrin derivatives are cyclic oligomers formed by seven units of glucose via α-(1,4)-linkages, having a toroidal shape with hydrophobic cavity and hydrophilic exterior. Due to this specific structure, they act as molecular hosts for a large variety of guest molecules, both polar and non-polar, through non-covalent interactions [14].

The aim of this study is to compare the anti-inflammatory activity of genistein with the one of genistein incorporated in two ramified beta-cyclodextrins: hydroxypropyl-;beta-cyclodextrin (HPBCD) and randomly–metylated-beta-cyclodextrin (RAMEB); cyclodextrin-genistein products were physical-chemically analysed by X-ray diffraction, thermal analysis and electronic microscopy in order to evaluate the formation of real inclusion complexes. The study intends to examine the correlation between the increased water solubility of genistein, due to complexation, and its anti-inflammatory activity on animal model.

## Results and discussion

Genistein-β-CD complexes were previously prepared, as reported in the literature [15], mainly through the insertion of the guest A-ring into the cyclodextrin cavity. The complexes of genistein with the natural beta-cyclodextrin proved a higher solubility and bioavailability compared to the drug alone [16]. Considering the rather low water solubility of natural β-CD, studies have been conducted on the complexation of genistein with derivatisedcyclodextrins [17]. To the best of our knowledge, no studies have been performed on the biological properties (i.e. anti-inflammatory activity) of genistein complexes with semisynthetic hydrophilic derivatives of β-CD.

Complexes were analysed by X-ray diffraction, differential scanning calorimetry (DSC) and scanning electron microscopy (SEM) in order to establish the true inclusion nature of the final product.

**Figure 2:**
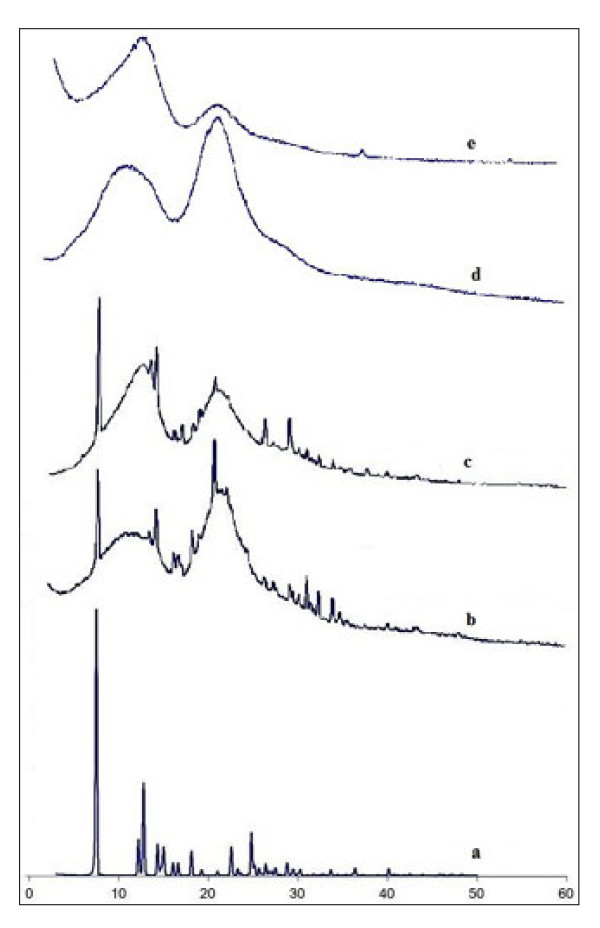
**X-ray diffraction patterns.** (**a**) genistein; (**b**) genistein: HPBCD; (**c**) genistein: RAMEB; (**d**) HPBCD; (**e**) RAMEB.

Figure 2 shows the diffraction patterns for genistein and its products with HPBCD and RAMEB. Genistein presents three major peaks at 2θ values of 7.51°, 12.78° and 24.81° with a range of smaller peaks accompanying them. Both cyclodextrins, HPBCD and RAMEB, show no crystallinity at all, having an amorphous structure. All the characteristic peaks of genistein are highly reduced in case of genistein 1:2 products indicating a partial amorphisation of the product. The peak at 2θ value of 18.14° shifts at 18.09° in HPBCD complex. The peak at 2θ value of 19.33° disappears completely at RAMEB complex and shifts at 19.23° in case of HPBCD complex, while the peak at 2θ value of 21.13° disappears in both cyclodextrin complexes. Two new peaks appear in case of HPBCD complex: at 2θ values of 14.83° and 20.97°, indicative of an interaction between the drug and the cyclodextrin, accompanied by intimate changes of the lattice level. There is also a shift of the peak at 2θ value of 28.84° at 28.76° and 28.79° in case of HPBCD and RAMEB complexes, respectively. The shifts of the peaks’ position and the appearance of new peaks in the XRD patterns of genistein products indicate the possibility of formation of a solid form with different properties or inclusion complexes.

DSC analysis was used in order to reveal the interaction between the drug and its host molecules, HPBCD and RAMEB. When guest molecules are trapped inside cyclodextrin cavities, their physical-chemical parameters (such as melting, boiling, sublimation points) change: they either disappear or shift to other temperatures [18].

**Figure 3:**
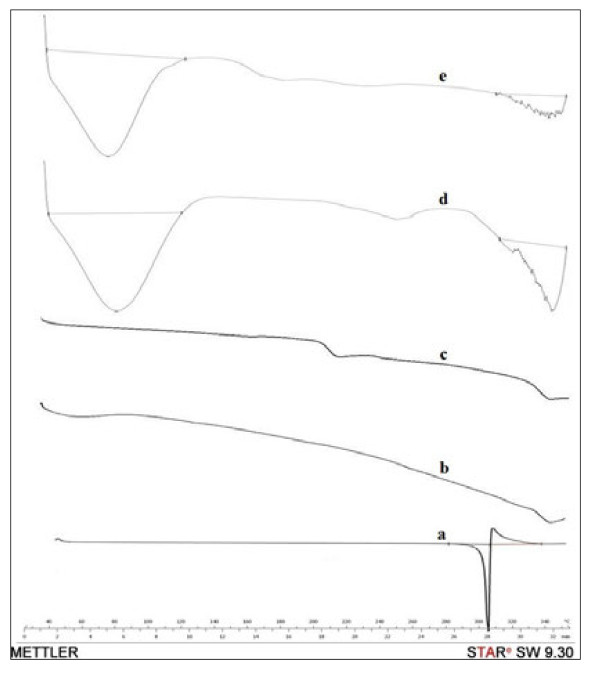
**DSC thermograms.** (**a**) genistein; (**b**) HPBCD; (**c**) RAMEB; (**d**) gen-HPBCD; (**e**) gen-RAMEB.

The DSC plot of pure genistein shows a crystalline state with a sharp endothermic peak at 303.66°C (ΔH =142.73 J·g^-1^) (Figure 3), which is attributed to its melting process. In the thermograms of both cyclodextrins no peaks were observed, confirming their amorphous structures; both cyclodextrins decompose above 300°C. For both cyclodextrins, a broad endothermic band was observed at around 100°C, which was related to the dehydration process. For the simple physical mixtures of pure genistein and cyclodextrins, thermograms showed a superposition of both components, with a partial amorphisation of the final products (not shown). The DSC curves of the 1:2 complexes of genistein with HPBCD and RAMEB, respectively, showed the complete disappearance of genistein fusion peak, indicating the formation of amorphous aggregates and the possibility of inclusion complex formation between the two substances. The complexes decompose above 300°C, similar to the pure cyclodextrins, a further evidence of the inclusion of genistein into the cyclodextrins’ matrices.

**Figure 4:**
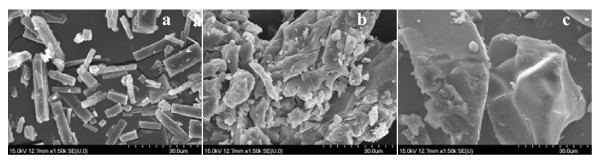
**SEM pictures.** (**a**) Gen; (**b**) Gen: HPBCD; (**c**)Gen: RAMEB.

The scanning electron microscopy images (Figure 4 a, b, c) show a difference between the morphology of the pure substance genistein compared to its two complexes with HPBCD and RAMEB, respectively. This analysis confirms the fact that the complexation took place and physical interactions were produced.

Genistein consists of large, pure crystals with a smooth surface and a regular prismatic shape. The size of tetragonal particles is between 5–30 μm. The SEM pictures reveal the morphological changes of the crystals after complexation: the regular, smooth surface disappeared, and, because of the interaction between the drug and cyclodextrins, following the preparation procedure, aggregation can be seen. The cyclodextrins presumably cover the surface of genistein. HPBCD and RAMEB are amorphous materials and complexation agents, therefore amorphisation and/or inclusion complexation of genistein is presumable. The regular shape of the drug is also affected; the amorphous HPBCD and RAMEB can be seen on the particle surface.

**Figure 5:**
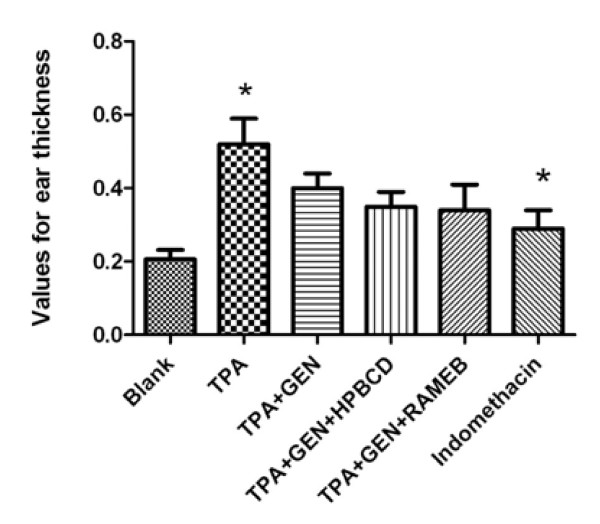
Differences between ear thicknesses in the six groups.

**Figure 6:**
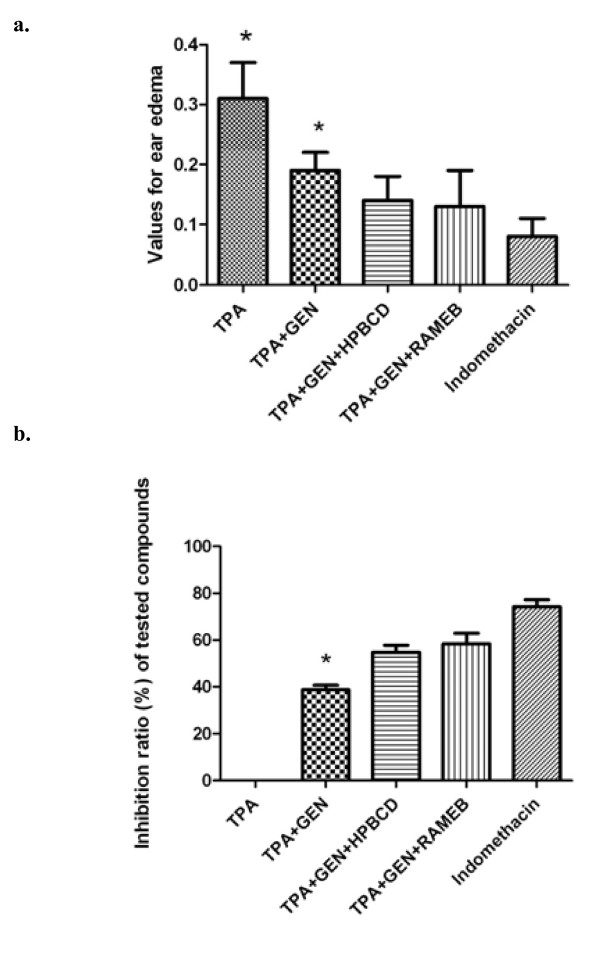
(a) Values for oedema after induction of inflammation and treatment; (b) Inhibitory ratio (%) of tested compounds.

In order to be able to compare the anti-inflammatory activity of soy isoflavonoid genistein, which has already been confirmed by previous studies [9-11], to the one of genistein incorporated in HPBCD and RAMEB, inflammation was induced using the mouse ear model for inflammation and ear thickness was measured. Results presented in Figure 5 indicate that the topical application of 10 μg 12-O-tetradecanoylphorbol-3-acetate (TPA) dissolved in 0.1 ml solvent (acetone : DMSO in a molar ratio 9:1) to both the inner and outer ear surfaces causes a significant increase in ear thickness after 24 hours: (0.52 ± 0.06 mm) compared to the one of mice in the blank group (0.21 ± 0.01 mm). Experimental groups treated with genistein alone or incorporated in CDs showed significantly reduced ear oedema compared to group B, where TPA alone was applied on the mice ear. In case of treatment with pure genistein, the average value for ear thickness decreased to 0.40 ± 0.03 mm. Smaller values, and therefore better anti-inflammatory response, was found in case of treatment with the complexes: genistein : HPBCD (0.35 ± 0.04 mm) and genistein : RAMEB (0.34 ± 0.06 mm). Indomethacin was used as control and the average value of ear thickness in this case was 0.29 ± 0.04 mm. After measuring ear thickness for each group, values for oedema and inhibitory rate were calculated (Figure 6 a, b). The average value for oedema induced by TPA alone was 0.31 ± 0.06 mm, IR = 0%, while the average value for oedema in case of treatment with genistein was 0.19 ± 0.03 mm, IR = 38.71%; for the CD-complexes of genistein, the measured average values of oedema were: 0.14 ± 0.04 mm, IR = 54.83% (HPBCD) and 0.13 ± 0.06 mm, IR = 58.06% (RAMEB). Indomethacin led to average values of 0.08 ± 0.04, IR = 74.19%. These results allow to draw the conclusion that genistein has an anti-inflammatory activity which becomes stronger for its complexes with hydrophilic CDs, but the values are smaller than in case of positive control using indomethacin.

**Figure 7:**
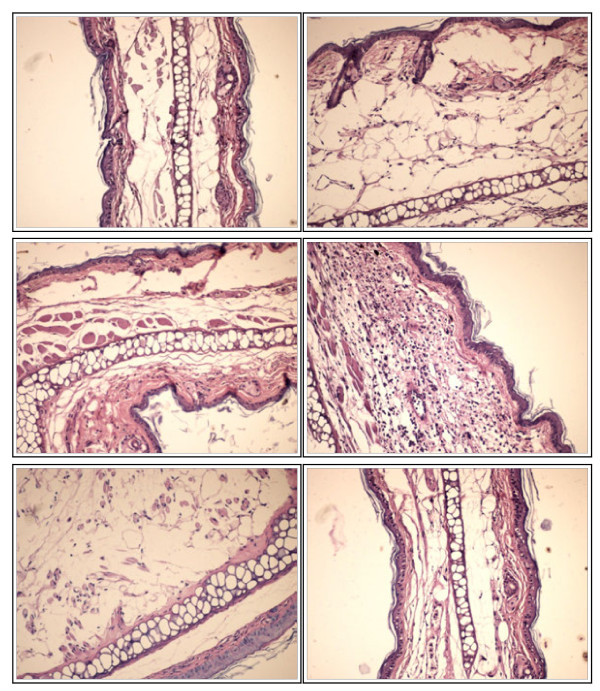
**Histological analysis. a**) Normal tissue - Group A (HE×200); **b**) Massive oedema of the superficial and deep dermis. Major destruction of the superficial dermis. Complete lyses of the muscular layer- Group B (HE×200); **c**) Massive oedema of the deep dermis. Partial destruction of the superficial dermis. Partial destruction of the underlying muscle cells- Group C (HE×200); **d**) Rich polymorphous inflammatory infiltrate that increases in thickness the sub epithelial dermis without inflammatory infiltrate in epithelial tissue-Group D (HE×200); **e**) Epithelial and cartilaginous tissue with marked edema Group E (HE×200); **f**) Loose stroma, oedematous, with low cellularity-Group F (HE×200).

After weighing the ears, morpho-pathological analyses were performed. Results can be seen in Figure 7 a - f. Figure 7a presents histological aspect of a normal tissue while Figure 7b lays out changes in the tissue after inflammation was induced: massive oedema of the superficial and deep dermis, major destruction of the superficial dermis and complete lyses of the muscular layer were detected. Acute inflammation occurs in two overlapping stages: vascular and cellular. Oedema, an established marker of acute inflammation is present due to an increased blood vessel wall permeability followed by plasma extravasation [19-21]. Neutrophils ar capable of direct lyses of muscle cell membranes through a superoxide-dependent mechanism [22,23]. Data suggest that topical application of 10 μg TPA dissolved in 0.1 ml solvent to the inner and outer surfaces of the ear is a reliable and easily reproducible model for acute inflammation in experimental animals [24-26]. When genistein was applied, Figure 7c, massive oedema of the deep dermis was still present but only a partial destruction of the superficial dermis and of the underlying muscle cells could be detected. Inhibition of TPA-induced inflammation might be associated with the genistein’s capacity to suppress the production of reactive oxygen species and other inflammatory mediators [3]. Results are consistent with the findings of other groups: Bandara et al. [10] underlined the anti-inflammatory activity of topical isoflavonoids, using UVB irradiation to induce inflammation in mouse skin. Huang et al., 2010 [27], noticed the positive effect of soy isoflavone extract from soybean cake for the protective effects against UVB-induced damage, including inflammation. An improved anti-inflammatory activity of genistein was detected when genistein was incorporated in the two β-CDs. In case of the complex genisten : HPBCD, Figure 7d, haematoxylin-eosin (H&E) analysis detected a rich polymorphous inflammatory infiltrate that increased in thickness the sub epithelial dermis without the presence of an inflammatory infiltrate in the epithelial tissue, while in case of the complex genistein : RAMEB, Figure 7e, H&E analysis detected an epithelial and cartilaginous tissue with marked oedema. Inclusion complexes of cyclodextrins with nonpolar drugs represent a topic of high interest in pharmaceutical research, for providing the possibility to increase the water solubility, stability and bioavailability of the guest drugs [14,17,28,29]. Genistein bioavailability can be increased by complexation with CDs [30]. Zhao et al. [31] found an enhanced bioavailability of soy isoflavones by complexation with two commercially available dendrimers.

For the control group, mice treated with indomethacin, Figure 7f, a loose stroma, oedematous, with low cellularity was detected. Indomethacin is a consecrated typical nonsteroidal anti-inflammatory compound [32,33].

**Figure 8:**
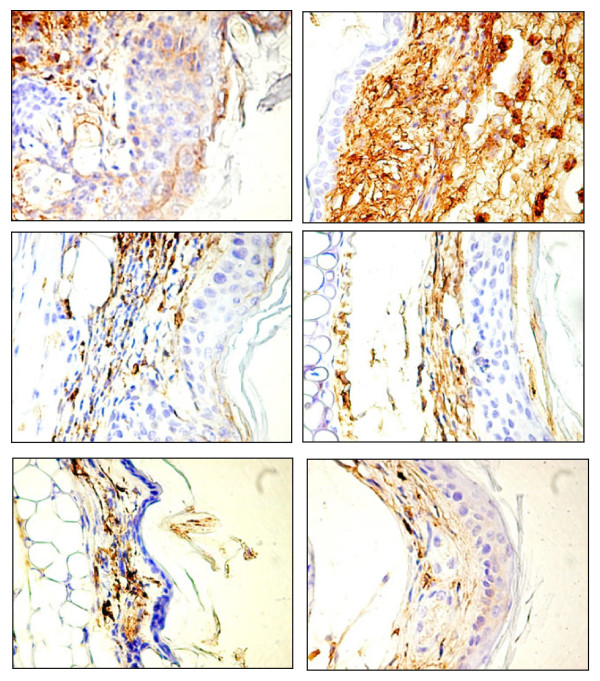
**Expression of the marker CD45 in the different groups (x 400).a**)- Group A; **b**)- Group B; **c**)- Group C; **d**)- Group D; **e**)- Group E; **f**)- Group F.

In order to observe some of the cellular events, more specific migration of leucocytes at the site of injury, expression of the marker CD45 was analysed (Figure 8 a-f). CD45, a transmembranar leukocyte-specific protein tyrosine phosphatase, is an abundant cell surface glicoprotein on lymphocytes. It exhibits multiple splicing isoforms and modulates the function of the haematopoietic cells. CD45 is also called the common leukocyte antigen [34,35]. Because inflammation is characterised by a transfer of plasma and leucocytes from the blood into the damaged tissue [36], CD45 is a good marker to differentiate the degree of inflammation. Acute inflammation is mediated by granulocytes, mainly neutrophils [37,38] while macrophages together with lymphocytes are involved in the production mechanism of chronic inflammation [39,40]. In this study one can observe the weak expression of CD45 in case of group A, blank, and group F, mice treated with indomethacin, a classical anti-inflammatory compound [32,33] in contrast with the strong expression of the marker in case of group B, mice on which TPA in acetone was administered. Due to an increased presence of leucocytes in this group, again one can confirm that TPA is a good inductor of inflammation. The expression of the marker is reduced in case of group C, mice treated with genistein, compared to group B, but higher than the expression in group F. This allows us to assert that genistein possesses anti-inflammatory proprieties, but milder than the ones of indomethacin. Regarding the presence of leucocytes in case of group D, mice treated with genistein incorporated in HPBCD, and group E, mice treated with genistein incorporated in RAMEB, their number is quite similar with slight increased values in case of group E. In both cases the expression is weaker than in case of group C, confirming again the findings that genistein incorporated in β-CDs presents a greater bioavailability, translated in a stronger anti-inflammatory effect.

## Conclusions

As conclusion, genistein can be reconsidered as an active anti-inflammatory phytocompound on C57BL/6 J animal model. Complexation of this active phytocompound with ramified β-CD derivatives was feasible and led to a stronger anti-inflammatory activity, proving to be a good method to improve the water solubility of genistein in order to obtain a better therapeutic response. These findings are of special interest since pharmaceutical formulations that produce increased drug concentration at the delivery site represent a better therapeutic option for patients.

## Methods

### Reagents

Genistein was purchased from Extrasynthese (France, purity >95%), hydroxylpropyl-beta-cyclodextrin (HPBCD) and randomly-metylated-beta-cyclodextrin (RAMEB) from Cyclolab Hungary, 12-*O-*tetradecanoylphorbol-13-acetate (TPA) from Sigma Aldrich, Germany. All substances were used as received.

### Preparation of complexes

Different methods were applied for the preparation of the inclusion complexes:

simple powder mixing, using a mortar and a pestle;

kneading with a 50% (w/w) water : ethanol solution until the bulk of solvent evaporated and a paste-type product was formed; the mixture was then dried at room temperature for 24 hours and put in the oven, at 105°C, for several hours until reaching constant weight. The final product was pulverized and sieved.

All the binary products were prepared using 1:2 genistein : CD molar ratio (Mw_Gen_ = 270,25, Mw_HPBCD_ = 1396, Mw_Rameb_ = 1303). The molar ratio of 1:2 was chosen in order to achieve a better water solubility for genistein.

### Differential scanning calorimetry (DSC)

The DSC measurements were made with a Mettler Toledo DSC 821^e^ thermal analysis system with the STAR^e^ thermal analysis program V9.1 (Mettler Inc., Schwerzenbach, Switzerland). Approximately 2–5 mg of genistein or its product was examined in the temperature range between 25°C and 350°C. The heating rate was 5°C min^-1^. Argon was used as carrier gas, at a flow rate of 10 l h^-1^ during the DSC investigation.

### Scanning electron microscopy (SEM) assay

The shape and surface characteristics of genistein and complex were visualized using a scanning electron microscope (Hitachi S4700, Hitachi Scientific Ltd., Japan). The samples were sputter coated with gold–palladium under an argon atmosphere using a gold sputter module in a high vacuum evaporator and the samples were examined using SEM set at 15 kV.

### X-ray diffraction

X-Ray-diffraction patterns were obtained on a Philips PW 1710 diffractometer, where the tube anode was Cu with *K*α = 1.54242 Å. The pattern was collected with a tube voltage of 50 kV and 40 mA of tube current in step scan mode (step size 0.035, counting time 1 s per step).

### Animal studies

*Ethical statement:* The work protocol followed all NIAH-National Institute of Animal Health rules: animals were maintained during the experiment in standard conditions: 12 h light–dark cycle, food and water *ad libitum*, temperature 24°C, humidity above 55%. The experiment was conducted according to the rules of the Ethical Committee of UMF “Victor Babes” Timisoara.

Animal studies were conducted on 8-week old C57BL/6 J female mice. Mice were purchased from Charles River (Sulzfeld, Germany). The number of mice included in the study was forty-eight (48), divided into six groups as follows:

group A: blank group

group B: mice on which TPA in acetone/DMSO was applied on the ear

group C: mice on which TPA in acetone/DMSO and genistein (30 minutes later) were applied on the ear

group D: mice on which TPA in acetone/DMSO and genistein : HPBCD 1:2 (30 minutes later) were applied on the ear

group E: mice on which TPA in acetone/DMSO and genistein : RAMEB 1:2 (30 minutes later) were applied on the ear

group F: mice on which TPA in acetone/DMSO and indomethacin (30 minutes later) were applied on the ear as control.

### Inflammation protocol

Inflammation was induced in both ears of each mouse by the topical application of 10 μg TPA dissolved in 0.1 ml acetone : DMSO in a molar ratio 9:1 to both the inner and outer ear surfaces. Thirty minutes after the application of TPA, the inner and outer surface of each ear was treated with 2 mg of genistein, or the equivalent quantity of 2 mg genistein corresponding from the complex Gen : HPBCD and Gen : RAMEB. The same quantity of indomethacin, 2 mg, was used as control. The solvent was acetone : DMSO in a molar ratio 9:1 and the quantity administered was 0.1 ml.[25,41] After 24 hours from the moment of application skin inflammation was induced, indicated by the increase of ear thickness on C57BL6/J mice. Ear thickness was measured with callipers, before treatment (value a) and 24 hours after TPA application (value b = TPA alone and value c = TPA + active substance). The experiment was repeated three times and results are expressed as mean ± standard deviation. The following values were also calculated:

Oedema X induced by TPA alone (b-a),

Oedema Y induced by TPA plus a sample (c-a),

Inhibitory rate (%) [(Oedema X-Oedema Y)/Oedema X] x 100.

After that, mice were killed by cervical dislocation and 6 mm^2^ diameter ear punch biopsies were collected and H&E analysis was carried out [24,26]. The Prism software package (Graph Pad Prism 4.03 for Windows) was used for data presentation. The experiment was repeated three times and results are presented as mean ± SD. Paired Student’s *t* tests was applied to evaluate statistical significance (∗, *p* < 0.05; ∗∗, *p* < 0.01; and ∗∗∗, *p* < 0.001).

### Histology

For the histological analysis, tissue samples (skin) were fixed in 10% formalin solution, embedded in paraffin and cut at 4 microns. Finally, deparaffinized, the samples were stained with H&E (hematoxylin-eosin) and microscopically analysed. Immunohistochemistry was performed by using CD45 antibodies against inflammatory cells from the dermis. After dewaxing and rehydration, the sections were incubated with CD45 for 30 minutes, and then, the antigen-antibody reaction was detected by using an avidin biotin system from Novocastra. Visualization of the final product was done by using 3,3’diaminobenzidine as chromogen. Counterstain was performed with Lille’s modified haematoxylin.

## Competing interests

Authors declare no competing interests.

## Authors’ contributions

CD(T) carried out the design of the study, participated in animal studies and drafted the manuscript. CS carried out preparation of cyclodextrin complexes, physico-chemical analysis and performed statistical analysis and final interpretation of results. EC and RA participated in physico-chemical analysis. SF and CP carried out the immunoassays. CD participated in the study’s design and coordination and helped to draft the manuscript. All authors read and approved the final manuscript.
